# Septic Shock and Severe AKI Caused by West Nile Virus in Southern France

**DOI:** 10.1016/j.ekir.2026.106544

**Published:** 2026-04-16

**Authors:** Anna Crepet, Mickaël Bobot, Esther Cortes, Raphaelle Klitting, Laura Pezzi, Nazli Ayhan, Xavier De Lamballerie, Guillaume Durand, Nadim Cassir

**Affiliations:** 1 Infectious Diseases Department, IHU-Méditerranée Infection, Marseille, France; 2 Nephrology Department, Centre de néphrologie et transplantation rénale, Assistance publique-Hôpitaux de Marseille, Hôpital de la Conception, Marseille, France; 3 Aix-Marseille Université, Inserm 1263, INRAE 1260, C2VN, Marseille, France; 4 CERIMED, Aix-Marseille Université, Marseille, France; 5 Intensive Care Department, Service de réanimation, Hôpital Privé La Casamance, Aubagne, France; 6 National Reference Center for Arboviruses, Inserm-IRBA, Marseille, France; 7 Virology Department, Unité des Virus Émergents: Aix-Marseille Université, Università di Corsica, IRD 190, Inserm 1207, Marseille, France; 8 Aix-Marseille Université, AP-HM, MEPHI, Marseille, France

## Introduction

West Nile virus (WNV) is a neurotropic orthoflavivirus primarily transmitted by *Culex* mosquitoes and rarely, through blood transfusion or organ transplantation. Birds constitute the main reservoir, whereas humans and horses are considered dead-end hosts.^1^ In 2025, 1112 locally acquired WNV cases were reported across 14 European countries, including 62 in France, 31% of which occurred in the Bouches-du-Rhône department in southern France.^2^

Approximately 80% of WNV infections are asymptomatic, whereas approximately 20% result in a febrile illness. Fewer than 1% of patients develop neuroinvasive disease, which carries a case fatality rate of approximately 10%.^1^ Severe cases may present with systemic illness requiring intensive care, even in the absence of neurological involvement.^3^ Reported risk factors for severe or atypical disease include advanced age, male patients, diabetes mellitus, cardiovascular disease, chronic kidney disease (CKD), and immunosuppression.S1^,^S2 Severe disease is often associated with cytopenia and multiorgan dysfunction, including acute kidney injury (AKI).^1^^,^S3 These features may occur in the absence of overt neurological involvement, contributing to diagnostic delay and misdiagnosis as bacterial sepsis.

The kidney is increasingly recognized as an important extraneurological target of WNV infection.^4^ WNV exhibits renal tropism in humans and animal models, frequently resulting in viruria. Animal models consistently identify the renal tubular epithelium (particularly proximal tubules, with occasional collecting duct involvement) as the primary target. AKI can occur in 9% of hospitalized cases of WNV infections.^5^ Acute lesions include tubular epithelial degeneration or necrosis, casts, occasionally responsible for transient proximal tubular dysfunction and focal or diffuse interstitial nephritis.S4^,^S5 Chronic infection may lead to low-grade interstitial inflammation progressing to fibrosis and tubular atrophy, leading to CKD.^6^ In addition, WNV infections seem to be more frequent in patients with CKD, and kidney transplant recipients.S6 We report on a case of WNV infection with severe systemic illness in the Bouches-du-Rhône department, southern France. Key teaching points of this case are presented in Table 1.

**Table 1:** Teaching points

Key message	Clinical implications
WNV infection may present as severe systemic illness with septic shock and multiorgan dysfunction even in the absence of early neurological symptoms.	Clinicians should consider WNV in patients with unexplained shock, cytopenias, and inflammatory syndrome during mosquito transmission seasons.
WNV demonstrates renal tropism, and acute kidney injury may occur as part of systemic infection.	Patients with severe WNV infection may require renal replacement therapy and should undergo renal follow-up
Detection of WNV RNA in urine may persist longer than in serum because of viral shedding from infected renal tubular epithelium.	Urine PCR can extend the diagnostic window, particularly in delayed presentations or critically ill patients.

PCR, polymerase chain reaction; WNV, West Nile virus.

## Case Presentation

In September 2025, a 63-year-old man developed fever, arthralgias, and fatigue, followed by dyspnea and oppressive chest pain, prompting admission to the emergency department. His medical history included noninsulin-dependent type 2 diabetes mellitus, previous pulmonary embolism, and coronary artery disease with stent placement.

**Figure 1:**
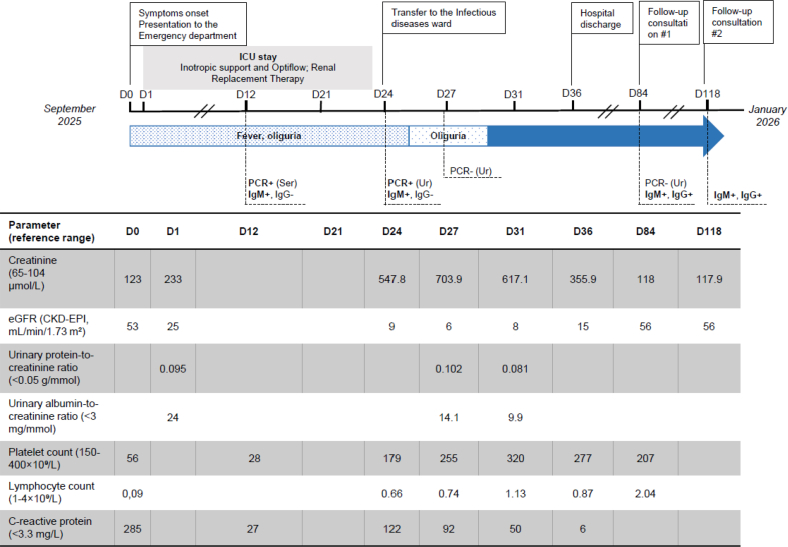
Clinical features and laboratory findings. CKD-EPI, Chronic Kidney Disease: Epidemiology Collaboration; D, day; eGFR, estimated glomerular filtration; ICU, intensive care unit; NA, not applicable; PCR, polymerase chain reaction; Ser, serum; Ur, urine.

Three days before presentation, he had returned from a 4-day trip to the southern coast of Spain (Catalonia region), where he did not recall mosquito exposure. At presentation, he was pyrexial but hemodynamically stable. Imaging was unremarkable, except for hepatomegaly with steatosis. Laboratory investigations revealed marked lymphopenia (0.09 G/l), thrombocytopenia (56 G/l), mildly elevated creatinine (123 μmol/l), and elevated inflammatory markers (C-reactive protein at 285 mg/l) (Figure 1).

Within 24 hours, the patient developed worsening respiratory failure, hyperlactatemia (4 mmol/l) and AKI, requiring intensive care unit admission for septic shock with multiorgan failure.

Over the following days, his condition further deteriorated, with agitation and requirement of high flow nasal oxygenation (Optiflow), vasopressor support with noradrenaline (1 μg/kg/min), dobutamine (10 μg/kg/min) and albumin 20%. He progressed to severe Kidney Disease: Improving Global Outcomes stage 3 anuric AKI, with a peak creatinine at 700 μmol/l, necessitating renal replacement therapy by continuous venovenous hemofiltration with Baxter Prismaflex machine (blood flow: 150 ml/min, fluid replacement volume: 35 ml/kg/h) using citrate as anticoagulant therapy. Urinalysis showed features consistent with severe acute tubular necrosis, including tubular proteinuria (urinary protein-to-albumin ratio: 0.129 g/mmol and urinary albumin-to-creatinine ratio: 33 mg/mmol) without urinary leukocyturia or hematuria. Lumbar puncture was not performed because of hemodynamic instability and transient altered mental status.

During his 3-week intensive care unit stay, he remained febrile. Blood cultures were negative, and extensive infectious workup, including polymerase chain reaction and serology for leptospirosis was negative. He received empirical broad-spectrum antibiotic therapy with piperacillin-tazobactam, clindamycin, and linezolid; linezolid was later replaced with vancomycin because of worsening thrombocytopenia. Renal replacement therapy was continued for 23 days and discontinued after progressive recovery of diuresis without fluid overload. At that time, proteinuria had improved (urinary protein-to-albumin ratio: 0.06 g/mmol; urinary albumin-to-creatinine ratio: 7 mg/mmol).

WNV infection was confirmed on day 12 post–symptom onset by positive polymerase chain reaction and serology (IgM positive and IgG negative). Viral RNA remained detectable in urine on day 24 post–symptom onset before clearance, and delayed IgG seroconversion was observed on day 84 post–symptom onset. Viral isolation and genome sequencing confirmed infection with WNV lineage 2, closely related to strains circulating in the Bouches-du-Rhône department (Figure 1, Supplementary Table S1, Supplementary Figure S1, Supplementary data).

At discharge, 1 month after symptom onset, the patient had persistently impaired kidney function (creatinine: 356 μmol/l, estimated glomerular filtration rate: 16 ml/min). At 3-month follow-up, creatinine improved to 118 μmol/l; however, stage 3A CKD persisted (estimated glomerular filtration rate: 54 ml/min), justifying the introduction of nephroprotective therapy, including ramipril 1.25 mg/d and empagliflozin 10 mg/d. Renal function remained stable at 4 months.

## Discussion

This case highlights severe WNV-associated septic shock with AKI requiring prolonged renal replacement therapy and resulting in CKD, in the absence of early neurological manifestations. The severity of multiorgan failure may mimic bacterial sepsis, underscoring the importance of considering arboviral infections in patients presenting with unexplained shock and cytopenia during transmission seasons (Table 1).

To our knowledge, this is the first reported case of severe AKI associated with septic shock owing to WNV infection in the Bouches-du-Rhône department.

Phylogenetic analysis suggests local acquisition, with viral genomes closely related to strains circulating in Bouches-du-Rhône between 2022 and 2023. However, limited data on circulating lineages in Spain, particularly in Catalonia, preclude definitive conclusions. In 2025, WNV cases in Spain were confined to central and western regions, with none reported in Catalonia (Supplementary data).^2^^,^S7^,^S8

Prolonged detection of WNV RNA in urine after clearance of viremia supports renal tropism and highlights the diagnostic value of urine polymerase chain reaction, particularly in delayed or severe presentations.S9^,^S10^,^S11 Consistent with previous human and animal studies, our patient showed delayed IgG seroconversion, reflecting impaired humoral responses and contributing to diagnostic uncertainty.^7^^,^S12 These findings support a combined molecular and serological approach to improve diagnostic sensitivity.S13^,^S14

Host factors, including diabetes and cardiovascular disease, may contribute to altered antiviral immune responses.S15 Experimental studies in type 2 diabetic models have demonstrated impaired viral clearance, delayed interferon responses, and reduced antibody production, leading to higher viral loads and delayed seroconversion.S16

Host genetic susceptibility and viral factors may also contribute to disease severity. Defects in innate immune pathways, particularly type I interferon signaling, have been associated with increased susceptibility to severe WNV infection.S17 Although the WNV lineage 1 has been more frequently linked to severe disease, recent data suggest that virulence is more strain-specific than lineage-specific, with some lineage 2 exhibiting comparable pathogenicity.S18^,^S19^,^S20

The occurrence of severe AKI requiring renal replacement therapy in this patient adds to growing evidence that WNV infection may lead to CKD.S21 Long-term renal follow-up should therefore be considered. In accordance with current 2012 Kidney Disease: Improving Global Outcomes and 2016 French recommendations, kidney function should be reassessed within 3 months after AKI.S22 Follow-up enables assessment of CKD progression risk and identification of factors associated with poor outcomes, including incomplete recovery, hypertension, albuminuria, and comorbidities such as diabetes mellitus. We recommend reassessment of kidney function, blood pressure, and albuminuria at 3 months, then annually.^8^

Management of WNV infection remains largely supportive, because no specific antiviral therapy is currently available. Passive immunotherapy with polyclonal and WNV-specific i.v. Ig has been explored in small case series, but has not demonstrated clear clinical benefit.^9^^,^S23

## Disclosure

All the authors declared no competing interests.

## Patient Consent

The authors declare that they have obtained written informed consent from the patients discussed in the report.

## Supplementary Material

Supplementary File (PDF) **Supplementary Data. Supplementary References. Figure S1.** Phylogeny of WNV lineage 2 highlighting sequences from the patient’s samples. **Table S1.** Next-generation sequencing of complete WNV genomes from the patient serum and urine.

## Footnotes

Supplementary File (PDF)

**Supplementary Data.**

**Supplementary References.**
**Figure S1.** Phylogeny of WNV lineage 2 highlighting sequences from the patient’s samples. **Table S1.** Next-generation sequencing of complete WNV genomes from the patient serum and urine.
