# Supplementary material for: Septic Shock and Severe AKI Caused by West Nile Virus in Southern France
Source: Kidney Int Rep. 2026 Apr 16;11(6):106544. doi: 10.1016/j.ekir.2026.106544 (PMC13188089; doi:10.1016/j.ekir.2026.106544)
Supplement: Supplementary File (PDF) — Supplementary Data. Supplementary References. Figure S1. Phylogeny of WNV lineage 2 highlighting sequences from the patient’s samples. Table S1. Next-generation sequencing of complete WNV genomes from the patient serum and urine. [file mmc1.pdf]

Supplementary data

Laboratory diagnosis of WNV infection

Blood samples obtained on day 12 post symptoms onset (pso) revealed a positive WNV PCR and serology results consistent with acute infection (IgM positive, IgG negative). WNV PCR in urine was positive on samples collected on day 24 pso and became negative by day 27 pso. IgG seroconversion was observed on serum collected 84 days pso, and virus neutralization test (VNT) was weakly positive. The PCR-positive serum and urine samples were successfully isolated on Vero E6 cells, with clear cytopathic effect (CPE) observed 7 days after inoculation.

Infection with WNV was further confirmed by next-generation sequencing of complete WNV genomes from the patient serum and urine (Pathoplexus accession numbers: PP_004J594.1, PP_004J5A2.1) **(Table S1)**. Phylogenetic inference using a maximum-likelihood approach showed that the virus strain responsible for the initial infection belonged to WNV lineage 2 and was related to strains sampled in the Provence-Alpes-Côte d'Azur region between 2018 and 2023 **(Figure S1)**.

RT-qPCR and serology

WNV RT-qPCR was realized using primers and probes (ref 001K-05424) and positive control (ref 001K-05425) provided by the European Virus Archive Marseille (EVAM, https://evam.european-virus-archive.com/) on the Panther Fusion system (HOLOGIC). The presence of anti-WNV antibodies was assessed using the EUROIMMUN Anti-WNV IgM and IgG ELISA assays. Virus neutralization test (VNT) was realized as previously described.

Virus sequencing at the National Reference Center for Arboviruses

WNV genomes were sequenced at the NRC using an amplicon-based sequencing approach with a set of 8 overlapping amplicons, previously used for WNV sequencing. These primers generate 35 overlapping amplicons across the full viral genome via two multiplexed PCR reactions **(Table S1)**.

Reverse transcription and amplification were performed using the SuperScript™ IV One-Step RT-PCR System with Platinum™ SuperFi DNA Polymerase (Thermo Fisher Scientific, USA). PCR reactions (25 µL final volume) consisted of 3 µL of nucleic acid extract, 1.25 µL of each primer (10 µM), 12.5 µL of 2× RT-PCR Master Mix, 0.5 µL of SuperScript IV RT mix, and 6.5 µL of RNase-free water. Thermocycling conditions were as follows: reverse transcription at 55 °C for 10 min, initial denaturation at 98 °C for 2 min, followed by 40 cycles of 98 °C for 10 s, 55 °C for 10 s, and 68 °C for 1 min 45 s, with a final extension at 68 °C for 5 min.

The size of PCR amplicons was verified by agarose gel electrophoresis. For each sample, an equimolar pool of all PCR products was generated and subsequently purified using the Monarch® PCR & DNA Cleanup Kit (New England Biolabs, USA). DNA quantification was performed using the Qubit® dsDNA HS Assay Kit with the Qubit 2.0 fluorometer (Thermo Fisher Scientific, USA). Purified amplicons were fragmented to an average size of ~250 bp using a Bioruptor® sonicator (Diagenode, Liège, Belgium). Fragmented DNA was then used for library construction with the Ion Plus Fragment Library Kit on the AB Library Builder™ System (Thermo Fisher Scientific), following the manufacturer’s protocol.

To ensure accurate equimolar pooling of barcoded libraries, quantitative real-time PCR was performed using the Ion Library TaqMan™ Quantitation Kit (Thermo Fisher Scientific). Equimolar pools of libraries were subjected to emulsion PCR, and the resulting products were loaded onto Ion 530™ chips using the Ion Chef™ Instrument (Thermo Fisher Scientific). Sequencing was conducted on the Ion S5™ System (Thermo Fisher Scientific) according to the manufacturer's instructions.

After demultiplexing, read data were analyzed with an in-house Snakemake pipeline. Reads were first trimmed using cutadapt (v4.4) to remove amplification primers and with trimmomatic (v0.39) to remove short and low-quality reads. Read alignment was achieved using BWA MEM (v0.7.17) using, as a reference, the best match identified by blasting (magicblast, v1.7.7) sequencing reads using a database of flavivirus sequences including reference sequences representative of the genetic diversity of WNV. Consensus sequences were called using the ivar (v1.3.1) consensus command with a minimum coverage depth of 50x.

Phylogenetic analysis

To ensure transparency and reproducibility, we have made the dataset publicly available as a SeqSet on the Pathoplexus platform (https://pathoplexus.org/), with the following DOI: 10.62599/PP_SS_67.2. The dataset includes all publicly available WNV lineage 2 genomes present in the Pathoplexus database as of 20 November 2025, with a sequence length greater than 8,500 nucleotides. Collection dates range from 1958 to 2023. Approximately 81% of sequences originate from Europe, 15% from Russia, and the remainder from Africa.

Sequences were trimmed to their coding regions (ORFs), filtered by size (only sequences with a length >8,500 nucleotides were selected), aligned using MAFFT (v7.511), and manually inspected in AliView (v1.0). We complemented this dataset by adding most recent sequences released from France. Phylogenetic relationships between public WNV genomes and sequences generated in this study were inferred using a maximum likelihood (ML) approach implemented in IQ-TREE (v1.6.12). The best-fit nucleotide substitution model was selected using ModelFinder, and branch support was evaluated with ultrafast bootstrap approximation (UFBoot2), using 1,000 replicates. All alignment and tree files for this study are available at: https://github.com/rklitting/WNV-APHM-2025.

Viral isolation on cell culture

PCR-positive serum and urine samples were diluted 1:8 in MEM (for Vero E6-green monkey kidney cells) or Leibovitz's L15 medium (for C6/36-*Ae. albopictus* cells) supplemented with 2.5% foetal bovine serum (FBS), 1% penicillin-streptomycin, 1% L-glutamine, 1% Kanamycin, and inoculated onto monolayers of Vero E6 and C6/36 cells in 6-well plates. After a one-hour incubation period (at 37°C with 5% CO_2 for Vero E6; 28°C without CO_2 for C6/36), the inoculum was replaced with specific growth media (MEM for Vero E6, L-15 for C6/36). Both media were supplemented with 7% heat-inactivated FBS, 1% penicillin–streptomycin, 1% L-glutamine, and 1% Kanamycin. Cell cultures were monitored daily for cytopathic effect (CPE). On day 5 post-inoculation, supernatants were harvested and analysed for WNV using extraction and RT-qPCR.

Figure S1. Phylogeny of WNV lineage 2 highlighting sequences from the patient samples.

Maximum-likelihood phylogeny reconstructed with IQ-TREE (1.6.12). The tree was inferred using a General Time-Reversible model (GTR) with empirical base frequencies and a FreeRate model (with 3 categories). Branch support was evaluated by ultrafast bootstrap approximation (UFBoot2), using 1,000 replicates. We used 607 sequences representative of the phylogenetic diversity within lineage 2, combined with those produced from the patient (in red) in this study. Sequences from France including those from Provence-Alpes-Côte d'Azur region (PACA) are shown in orange, sequences from Italy are shown in blue and those from Switzerland are shown in light grey. Nodes with a bootstrap support above 95 are shown with a black circle.

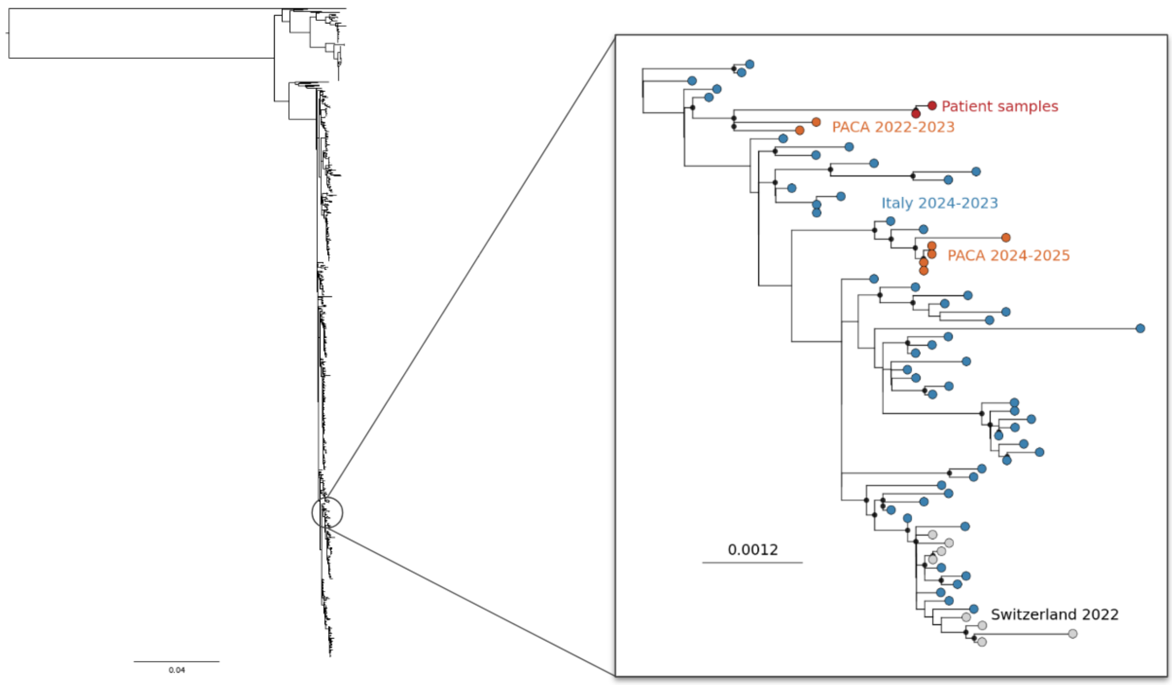

Table S1. Next-generation sequencing of complete WNV genomes from the patient serum and urine

Primer_name	Sequence	Pool
WNV_L2_1_LEFT	GAGGATTAATAACAATTAACACAGTGCGA	1
WNV_L2_1_RIGHT	AGCAAGATAGTAAAGCCCGCTG	1
WNV_L2_2_LEFT	TAGGAACTCTGACCAGTGCCAT	2
WNV_L2_2_RIGHT	TGTCTGGACAGTCAGAGACCTT	2
WNV_L2_3_LEFT	CTGCTGGTGCACGAAATCATCT	1
WNV_L2_3_RIGHT	TTATGGTCACACAGCTATCGCC	1
WNV_L2_4_LEFT	TGCTTGGGAATGAGTAACAGAGAC	2
WNV_L2_4_RIGHT	TGGCGACCTCATACTTGATGTTC	2
WNV_L2_5_LEFT	ATTGACACATGCGCGAAGTTTG	1
WNV_L2_5_RIGHT	TCAAACTCCATCAGTGTTTCCCG	1
WNV_L2_6_LEFT	CACCGAGAGTGGTTTATGGATCTG	2
WNV_L2_6_RIGHT	GTGAGGTCATTCAGGGAAGCTAC	2
WNV_L2_7_LEFT	CGGTGGTGTTGGAACTGCAATA	1
WNV_L2_7_RIGHT	CCCTCCAAAGAGTGATCTAAAAGCT	1
WNV_L2_8_LEFT	CTTGGAGACACTGCTTGGGATT	2
WNV_L2_8_RIGHT	CTCCTTCTGCATGTGCTTTCTGA	2
WNV_L2_9_LEFT	GCCTGGATGGATCGTTACAAGT	1
WNV_L2_9_RIGHT	GTCTTCTACCTCCATGCTGTTCC	1
WNV_L2_10_LEFT	AACACCTTTGTCATCGACGGTC	2
WNV_L2_10_RIGHT	CTGAGTTTTGTACCCTGGTCTCC	2
WNV_L2_11_LEFT	GTGGGGTGATGGAGTTTTGGAA	1
WNV_L2_11_RIGHT	CAGAAGGCCCAACTGAAAAGGA	1
WNV_L2_12_LEFT	TGGAATGGAAATTCGACCCACG	2
WNV_L2_12_RIGHT	CCATTTGAAAGAAAGCAGCTGCA	2
WNV_L2_13_LEFT	CAGTCTTTCTGGTGGCTTCCTT	1
WNV_L2_13_RIGHT	CCCAGCTGCAAGTATCATTGGA	1
WNV_L2_14_LEFT	GAGTTGGAAGCCTCATCAAAGAAAAA	2
WNV_L2_14_RIGHT	TGAAAATTTCCATCATCATCCAGCC	2
WNV_L2_15_LEFT	CGGCTGATATTACTTGGGAGAGTG	1
WNV_L2_15_RIGHT	TCATGAGAGCAGCTCCCTTAGT	1
WNV_L2_16_LEFT	TTGGCAGTTACCAAGCTGGAG	2
WNV_L2_16_RIGHT	CACAATGGCGCTTATGTATGAACC	2
WNV_L2_17_LEFT	TCAGGTTCCCCCATTGTGGATA	1
WNV_L2_17_RIGHT	AGACATCAGCCTGTGTGTGAGA	1
WNV_L2_18_LEFT	TACCAAACCTCAGCAGTGCAC	2
WNV_L2_18_RIGHT	GCAGACAGAGGGCAATCTCATT	2
WNV_L2_19_LEFT	GGAACACTGGATATGAATGGATAACTG	1
WNV_L2_19_RIGHT	TCATCCTCATTTGTGTGCCCTC	1
WNV_L2_20_LEFT	GAAGGTGATGGAAGAGTCATCCTG	2
WNV_L2_20_RIGHT	GTGTTGGTTCGAGGTCCATCAA	2
WNV_L2_21_LEFT	CAGTCTGGCTCGCTTACAAAGT	1
WNV_L2_21_RIGHT	AAGCTATTGTCTGAAGGGCGTC	1
WNV_L2_22_LEFT	TTGGACACGATGTATGTGGTGG	2
WNV_L2_22_RIGHT	CATTCTTGGTCTTGTCCAGCCA	2
WNV_L2_23_LEFT	CAACCAGCTTGCTGTGTTCTTG	1
WNV_L2_23_RIGHT	AGTCACAGTCACAGTCAGGGT	1
WNV_L2_24_LEFT	CTTTTGTGGACGTTGGTGTGTC	2
WNV_L2_24_RIGHT	CATTCCACACTGAACTAGCACCA	2
WNV_L2_25_LEFT	AGAACTGTCAGAGAGGCTGGAA	1

WNV_L2_25_RIGHT	GCCTTTCCACTAACCACCGTAA	1
WNV_L2_26_LEFT	ATGCCAGGAGAGAGGGAAACAT	2
WNV_L2_26_RIGHT	CAGTCTTCCACCATCTCCAAGAC	2
WNV_L2_27_LEFT	CACACTGCTCTGTGACATTGGA	1
WNV_L2_27_RIGHT	TTGAGGAGAGGCTTCCCTACTG	1
WNV_L2_28_LEFT	AAGAAAACATGGAAGGGACCCC	2
WNV_L2_28_RIGHT	TCGTGGTCTCATTGAGGACGTA	2
WNV_L2_29_LEFT	CTCCTTTCGGTCAACAACGAGT	1
WNV_L2_29_RIGHT	CCCCTAGCCACATGAACCAAAT	1
WNV_L2_30_LEFT	ACCTGCATCTACAACATGATGGG	2
WNV_L2_30_RIGHT	GGCCTCATCACTTTCACGACTT	2
WNV_L2_31_LEFT	CGAAGGTTCTTGAACTGCTGGA	1
WNV_L2_31_RIGHT	GGACCTTTGACATGGCATTTAGG	1
WNV_L2_32_LEFT	GGTGATGACTGCGTGGTGAA	2
WNV_L2_32_RIGHT	GAACCCAGTTGACAGGCACA	2
WNV_L2_33_LEFT	GGCTGTTGCTTTATTTTCACCGT	1
WNV_L2_33_RIGHT	GTGTCTTCATACCTCCTCAGGGA	1
WNV_L2_34_LEFT	TGGCAATCAATCAGGTCCGTTC	2
WNV_L2_34_RIGHT	ACACTGGGCTTTGAGGCTAAAG	2
WNV_L2_35_LEFT	GCTGTATTGAGTAGTTGTATAGTTGTAGTG	1
WNV_L2_35_RIGHT	GGTTAACACCATAGCCAGGGTT	1

Supplementary References:

S1. Madran B, Kaçar S, Douruk et al. Clinical spectrum, outcomes, and risk factors of West Nile virus infection: A systematic review and meta-analysis, *International Journal of Infectious Diseases.* 2026, doi: 10.1016/j.ijid.2026.108476

S2. Snyder RE, Cooksey GS, Kramer V, Jain S, Vugia DJ. West Nile Virus–Associated Hospitalizations, California, 2004–2017. *Clinical Infectious Diseases*. 2021;73(3):441-447. doi:10.1093/cid/ciaa749

S3. Brener ZZ, Harbord NB, Zhuravenko I, et al. Acute renal failure in a patient with West Nile viral encephalitis. *Nephrology Dialysis Transplantation*. 2006;22(2):662-663. doi:10.1093/ndt/gfl599

S4. Karamani P, Souroullas G, Nagash A et al. West Nile Virus Neuroinvasive Disease Presenting with Acquired Fanconi Syndrome, Which Resolved on Recovery. *European Journal of Case Reports in Internal Medicine* 2025; 12(11) doi: 10.12890/2025_005787

S5. Garcia-Tapia D, Hassett D, Mitchell W et al. West Nile virus encephalitis: sequential histopathological and immunological events in a murine model of infection. Journal of Neurovirology 2007; 13(2) doi: 10.1080/13550280601187185

S6. Aziz F, Saddler C, Jorgenson M et al. Epidemiology, management, and graft outcomes after West Nile virus encephalitis in kidney transplant recipients. *Transplant Infectious Disease* 2020; 22(4), doi: 10.1111/tid.13317

S7. Busquets N, Laranjo-González M, Soler M, et al. Detection of West Nile virus lineage 2 in North-Eastern Spain (Catalonia). *Transbound Emerg Dis*. 2019;66(2):617-621. doi:10.1111/tbed.13086

S8. García-Cervera C, Iftimie SM, Martínez MJ, et al. Report on the first two confirmed autochthonous cases of West Nile virus encephalitis in Catalonia, Spain. *Infect Dis (Lond)*. 2023;55(11):798-802. doi:10.1080/23744235.2023.2236703

S9. Barzon L, Pacenti M, Franchin E, et al. Isolation of West Nile virus from urine samples of patients with acute infection. *J Clin Microbiol*. 2014;52(9):3411-3413. doi:10.1128/JCM.01328-14

S10. Pacenti M, Sinigaglia A, Franchin E, et al. Human West Nile Virus Lineage 2 Infection: Epidemiological, Clinical, and Virological Findings. *Viruses*. 2020;12(4):458. doi:10.3390/v12040458

S11. Papa A, Testa T, Papadopoulou E. Detection of West Nile virus lineage 2 in the urine of acute human infections: West Nile Virus in Urine. *Journal of Medical Virology* 2014; Dec;86(12):2142-5. doi: 10.1002/jmv.23949

S12. Saxena V, Xie G, Li B, et al. A hamster-derived West Nile virus isolate induces persistent renal infection in mice. *PLoS Negl Trop Dis*. 2013;7(6):e2275. doi:10.1371/journal.pntd.0002275

S13. Xiao SY, Guzman H, Zhang H, Travassos da Rosa AP, Tesh RB. West Nile virus infection in the golden hamster (Mesocricetus auratus): a model for West Nile encephalitis. *Emerg Infect Dis*. 2001;7(4):714-721. doi:10.3201/eid0704.010420

S14. Tonry JH, Xiao SY, Siirin M, Chen H, Da Rosa APAT, Tesh RB. Persistent shedding of west nile virus in urine of experimentally infected hamsters. *The American Journal of Tropical Medicine and Hygiene*. 2005;72(3):320-324. doi:10.4269/ajtmh.2005.72.320

S15. Saiz JC, Martin-Acebes M, Blasquez A et al. Pathogenicity and virulence of West Nile virus

revisited eight decades after its first isolation. Virulence 2021; 12(1) doi: 10.1080/21505594.2021.1908740

S16. Kumar M, Roe K, Nerurkar P et al. Impaired virus clearance, compromised immune response and increased mortality in type 2 diabetic mice infected with West Nile virus. PloS One 2012; 7(8) doi: 10.1371/journal.pone.0044682

S17. Gervais A, Rovida F, Avanzini MA et al. Anti-type I IFNs auto-Abs in WNV encephalitis. *Journal of Experimental Medicine.* 2023; 220(9), doi: 10.1084/jem.20230661

S18. Botha E, Markotter W, Wolfaardt M et al. Genetic determinants of virulence in pathogenic lineage 2 West Nile virus strains. Emerging Infectious Diseases 2008-02(14) doi: 10.3201/eid1402.070457

S19. Byas AD, Ebel GD. Comparative Pathology of West Nile Virus in Humans and Non-Human Animals. *Pathogens*. 2020; 9(1):48, doi: 10.3390/pathogens9010048

S20. Venter M, Myers TG, Wilson MA, et al. Gene expression in mice infected with West Nile virus strains of different neurovirulence. *Virology*. 2005;342(1):119-140. doi:10.1016/j.virol.2005.07.013

S21. Hansen M, Nolan M, Gorchakov R et al. Unique cytokine response in west nile virus patients who developed chronic kidney disease: a prospective cohort study. Viruses 2021; 13(2) doi: 10.3390/v13020311

S22. Ichai C, Vinsonneau C, Souweine B et al. Insuffisance rénale aiguë en périopératoire et en réanimation (à l'exclusion des techniques d'épuration extrarénale). *Anesthésie & Réanimation* 2016, 2(3) doi: 10.1016/j.anrea.2016.04.001

S23. Gnann JW, Agrawal A, Hart J, et al. Lack of Efficacy of High-Titered Immunoglobulin in Patients with West Nile Virus Central Nervous System Disease. *Emerg Infect Dis*. 2019;25(11):2064-2073. doi:10.3201/eid2511.190537
